# Nutritional status of young children born with low birthweight in a low resource setting: an observational study

**DOI:** 10.1186/s12887-023-04356-9

**Published:** 2023-10-19

**Authors:** Flaviah B. Namiiro, Anthony Batte, Joseph Rujumba, Nicolette Nabukeera-Barungi, Violet O Kayom, Ian G. Munabi, Robert Serunjogi, Sarah Kiguli

**Affiliations:** 1 Department of Paediatrics & Child Health, Mulago National Referral Hospital, Kampala, Uganda; 2 Child Health and Development Center, College of Health Sciences, Makerere University, Kampala, Uganda; 3 Department of Paediatrics & Child Health, Makerere University College of Health Sciences, Kampala, Uganda; 4 Department of Anatomy, Makerere University College of Health Sciences, Kampala, Uganda; 5 Makerere University-John Hopkins University Institute, Kampala, Uganda

**Keywords:** Low birthweight, Nutrition, Stunting, Wasting, Underweight

## Abstract

**Objective:**

Every year, an estimated 20 million babies are born with low birthweight and this number is increasing globally. Survivors are at risk of lifelong morbidities like undernutrition. We assessed the growth and nutritional status for children born with low birthweight at Mulago Hospital, Uganda.

**Methods:**

We conducted a cross sectional study to describe the nutritional status of children aged between 22 and 38 months and born weighing ≤ 2000 g. Anthropometric measurements; weight for height, height for age and weight for age z-scores were generated based on the World Health Organization standards to define wasting, stunting and underweight respectively. Data was collected using a structured questionnaire and analysis was done using STATA version 14.

**Results:**

Of the 251 children, 129 (51.4%) were male, mean age was 29.7 months SD 4.5) and maternal mean age was 29.9 (SD 5.3). A total of 101(40.2%) had normal nutritional status. The prevalence of wasting, underweight and stunting were: 8 (3.2%), 36 (14.4%) and 106 (42.2%) respectively.

**Conclusion:**

Six of ten children born with low birthweight were at risk of undernutrition in early childhood: underweight and stunting were higher than the national prevalence. Targeted interventions are needed for children with very low birth weight.

## Background

Low birthweight is defined by the World Health organization (WHO) as weight at birth less than 2500 g. It is estimated 15–20% of all births globally are low birthweight (LBW), accounting for 20 million births annually [1]. Estimates of LBW vary across regions and within countries although majority occur in low- and middle-income countries (LMIC). More than 60% babies with LBW are born in Asia and sub Saharan Africa, with rates of 28% and 13% respectively [2]. Data on LBW remain limited in many LMICs as many births occur in homes or in facilities where birthweights are not taken or records are unreliable [3].

LBW is complex and may occur due to restricted fetal growth or preterm birth (born before 37 weeks of gestation), and/ or an overlap between the two [4]. In the current study, we focus on the birthweight categorized as low birthweight less than 2500 g, very low birthweight (VLBW) less than 1500 g and extreme low birthweight (ELBW) less than 1000 g. LBW is a significant public health issue associated with short- and long-term health consequences [3, 5, 6]. Health complications increase with reducing birthweight. Majority of the heavier babies (> 1500 g) will survive with minimal healthcare or no need for neonatal intensive care [3, 7]. LBW is a predictor of prenatal mortality and morbidity and has been found to increase risk for noncommunicable diseases such as diabetes, cardiovascular diseases and malnutrition among survivors extending to early childhood and adulthood [5, 8, 9].

Children born with LBW are at risk of growth and nutritional deficits. Regular assessment through follow-up and appropriate interventions to improve their outcome throughout their life course is crucial [10–12]. At 2–3 years of age, children born with LBW are expected to have caught-up on the growth curve with their normal birthweight counterparts [13, 14]. However, some studies have reported that at this age some children have nutritional deficits which may persists later in life [15].

Undernutrition is a known global burden affecting 165 million children below 5 years of age [16, 17] and those born with LBW are at higher risk. Stunting, wasting and underweight are established indicators for the nutritional status of infants and children, indicating their overall health and growth status. Cut-off references in public health are shown in Table 1. Stunting expressed as height-for-age is a chronic marker of nutritional deficit. Underweight expressed as weight-for-age and wasting as weight-for-height are acute markers of nutritional deficit [18]. Normal growth is defined by anthropometric measurements for age and sex with weight and length/height z-scores > -2 SDs of the reference population [19, 20].

**Table 1:** Cut-off values for public health significance

Indicator	Prevalence cut-off values for public health significance
Underweight	< 10%: Low prevalence 10–19%: Medium prevalence 20–29%: High prevalence ≥ 30%: Very high prevalence
Stunting	< 20%: Low prevalence 20–29%: Medium prevalence 30–39%: High prevalence ≥ 40%: Very high prevalence
Wasting	< 5%: Acceptable 5–9%: Poor 10–14%: Serious ≥ 15%: Critical

***Reference: WHO: 1995***

There is scarcity of data on early childhood nutritional status of children born with LBW in our setting. Despite the high prevalence of LBW in Uganda [21], minimal efforts exist at national level towards nutritional and growth monitoring beyond the neonatal period for this high risk population. We sought to evaluate the growth and nutritional status for children aged 22 to 38 months born with LBW in a low resource setting. The study provided important information on early childhood nutritional status for children born with LBW at Mulago hospital national referral in Kampala, Uganda.

## Methods

We conducted a cross sectional study for children born with LBW at the follow-up clinic at Mulago hospital, Kampala. Mulago hospital is also the training institution for Makerere University, College of Health Sciences. It serves mainly the urban and peri-urban population of Kampala the capital city and those referred from other facilities around the country. The clinic is run twice a week for children discharged from the neonatal unit whose birthweight was < 2500 g and /or born < 37 weeks of gestation. The follow-up schedule for the infants is as follows: they are seen in the clinic every fortnight until they gain a weight of ≥ 2500 g, then the interval for follow-up is every two to three months until 18 months of corrected age or two years of life. Services at the follow-up clinic are free of charge and include nutritional education, growth and development assessment of the infants. These are provided by a pediatrician, resident doctor and a nurse. On average, 30 infants are seen every week although nearly 300 neonates are admitted to the neonatal unit every month. Less than 50% of children attended the follow-up clinic for longer than a year (2016 hospital records).

**Fig. 1:**
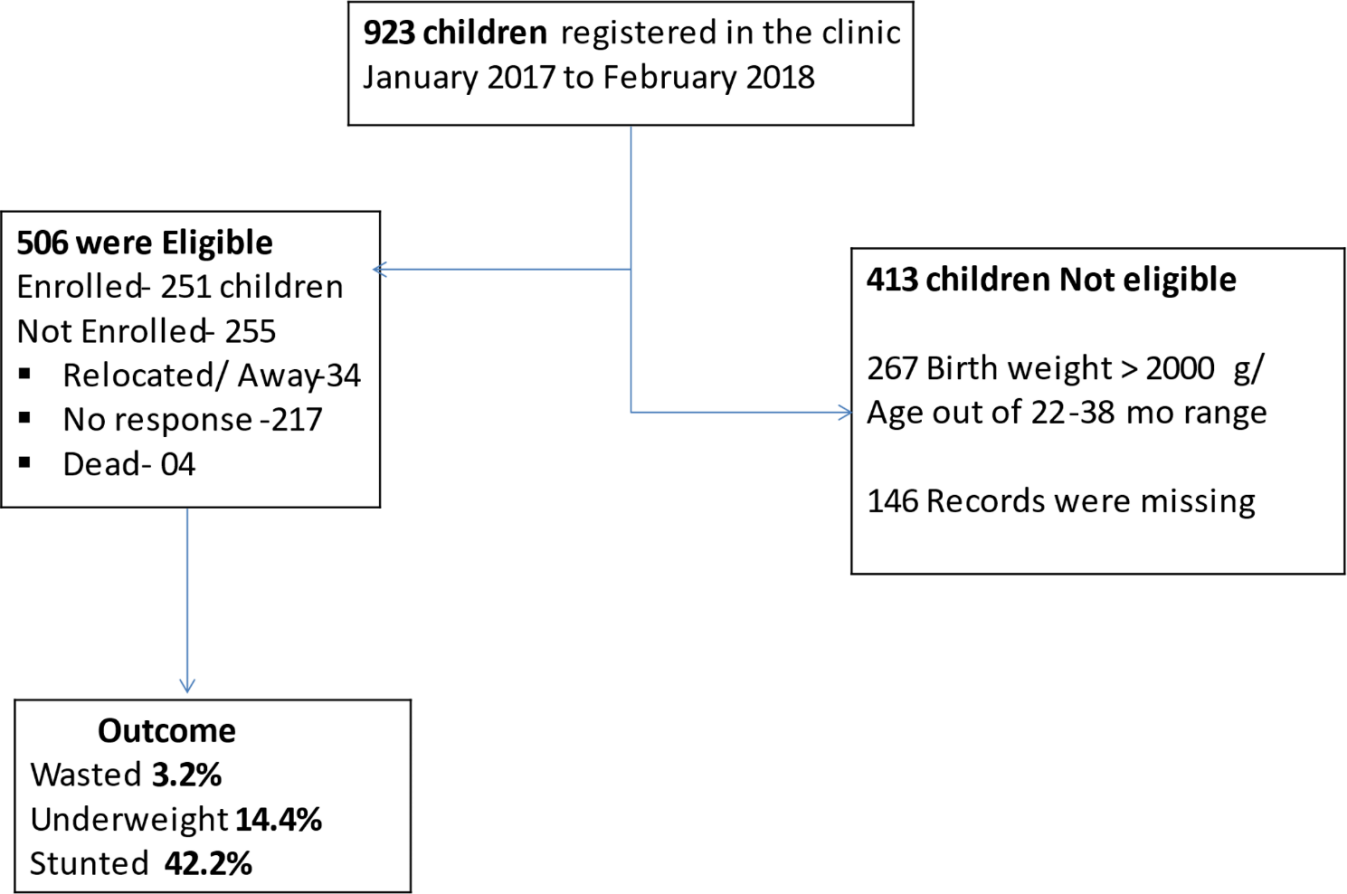
Flow chart

From November 2019 to February 2020, 251 children whose chronological age was 22–38 months at the time of the study, and had birthweight ≤ 2000 g (they are most likely to be preterm with more health problems) were included. Exclusion criteria were those with congenital anomalies and those hospitalized at the time of the study. Participants were identified from the pediatric outpatient/clinic records for those who ever attended between January 2017 to February 2018. A list of 506 eligible children was made, summarized in the flow chart, Fig. 1. We consecutively called up every caretaker when the initial method of calling every second mother did not yield our desired sample size. There was no response to some of the telephone calls made to the caretakers after three attempts. There were also some incomplete or wrong telephone numbers, and other numbers were out of service at the time of the study. A few caretakers/mothers were not able to come back to the clinic despite responding to our call.

### Data collection and study measurements

At enrolment, research assistants (nurses) obtained both infant and maternal demographics (age, sex, birth weight and socioeconomic characteristics). Other data collected included: mode and type of delivery, estimated distance from hospital, duration in hospital, duration of exclusive breastfeeding and duration in the follow up clinic. Data was collected using a structured questionnaire from the patients’ clinic records, immunization cards and discharge forms.

Anthropometric measurements: weight and height/length were taken following World Health Organizations (WHO) standard procedures [22]. The weight was taken using a digital portable SECA® weighing scale (Seca 813, Hamburg, Germany) corrected to the nearest 100 g, with the child wearing light clothing and bare feet.

Height/Length was measured using an infant length board (Infant/Child Shorr-Board®, Maryland, USA). Length was measured for children less than two years of age (i.e. up to and including 23 months) while in supine position. Mid-arm upper circumference was taken using color coded tapes (Child 11.5 red/pac-50, UNICEF), both to the nearest 1 mm. Triple measurement for weight, length/height and MUAC were taken and an average obtained.

Anthropometric z-scores for weight-for-height/length (WHZ), weight-for-age (WAZ) and height/length-for-age (HAZ) were computed using WHO Anthro version 3.2.2 [20]. Mother’s weight and height were also obtained and the body mass index (BMI) was calculated by dividing the weight in kilograms by the height in meters squared.

### Analysis and data management

Data was analyzed using STATA version 14 statistical software (StataCorp. 2017 College Station, TX: StataCorp LLC). The WHO Anthropometrics software was used to convert height, weight and age measurements to height-for-age z-scores (HAZ), weight-for-height z-scores (WHZ) and weight-for-age z-scores (WAZ) which were used to classify stunting, wasting and underweight respectively when z-scores were less than minus 2 SD. The presence of stunting, underweight and wasting among the children were performed using the WHO classification, Table 1, for assessing severity of malnutrition by prevalence ranges among children under-5 years of age [20]. We generated frequencies and percentages of children stunted, wasted and underweight.

## Results

### Baseline characteristics of the children and their mothers/caretakers

We enrolled 251 children mean age was 29.7 months (SD 4.5) and 51.4% were male. Most of the participants were born by spontaneous vaginal delivery 177 (70.5%) and majority were singletons 179 (71.3%). There were two sets of triplets while the other multiple births were twins. Children with birth weight ≤ 1500gm were 104 (41.6%) and those > 1500 were 146 (58.4%). The median duration of hospital stay post- delivery was 12 days (IQR 7–18). The anthropometric measurements for 236 mothers were included for analysis because 15 children were accompanied by other caretakers (10 fathers, 2 grandparents and one aunt). The summary of the baseline characteristics for the children and their mothers/caretakers are summarized in Table 2. Most mothers were in the informal type of employment, few had attained college or skilled training to translate into formal employment due to the high school dropout.

**Table 2:** Baseline characteristics for the children and caretakers

Variable		Frequency	Percentage %
Sex of the child			
Male		129	51.4
Female		122	48.6
Age of the child			
22–30 months		141	56.2
31–38 months		110	43.8
Mode of delivery			
Caesarean section		74	29.5
Vaginal delivery		177	70.5
Type of delivery			
Multiple*		72	28.7
Singleton		179	71.3
Birth weight			
≤1500 g		104	41.6
>1500 g		146	58.4
Child EBF			
Yes		165	65.7
No		86	34.3
Duration of hospital stay			
1–7 days		134	53.4
8–14 days		98	39.0
15–28 days		19	7.6
Mother’s Age			
≤25 years	50		19.9
26-30years	103		41.1
> 30 years	98		39.0
Estimated Distance from home to hospital			
< 5Km	28		11.1
5-15 km	212		84.5
≥ 15Km	11		4.4
Mother’s Education level			
Primary below/none	51		20.3
Secondary/tertiary level	200		79.7
Mother’s Marital status			
Married / co-habiting	199		79.3
Single / divorced/ separated	52		20.7
Mother’s Employment status			
Employed	164		65.3
Not employed	87		34.7
Mother with social support			
Yes	224		89.6
No	26		10.4
Number of children in family			
1–2 children	111		44.9
3–4 children	87		35.2
>5 children	49		19.9
Mother’s BMI			
< 18.5 (Underweight)	1		0.4
18.5 - ≤ 25 (Normal Range)	106		42.2
25.0- <30 (Overweight)	78		31.1
≥ 30 (Obese)	66		26.3

EBF: Exclusive Breastfeeding for first 6 months of life, *Multiple deliveries include 2 sets of children who were delivered as triplets and others were delivered as twins. BMI: Body Mass Index

### Growth and nutrition status of the children

Of the children studied, 101 (40.2%) had normal anthropometric measurements for their age and sex based on the reference population [20]. The prevalence for wasting (weight for height z-score <-2SD) was 8 (3.2%), underweight (weight for age z-score <-2 SD) 36 (14.4%) and the prevalence of stunting (height for age z-score <-2) was 106 (42.2%). There were more boys stunted, 64 out of 106 participants compared to girls (p = 0.001). The relationship of child and maternal characteristics with underweight and stunting are summarized in Tables 3, 4, 5 and 6. None of the children reported a recent acute illness (history within two weeks).

**Table 3:** Relationship of child characteristics with underweight

Variables	Nutritional status		COR 95% Cl	P value	aOR 95% CI *	P value
	Underweight (W/A Z-score <-2) (n = 35) (f, %)	Not Underweight W/A Z-score ≥-2) (n = 216) (f, %)				
Sex of the child						
Male	17(48.6)	112(51.9)	0.87 (0.43, 1.79)	0.719		
Female	18(51.4)	104(48.2)				
Age of the child						
22–30 months	17(48.6)	124(57.4)	1			
31–38 months	18(51.4)	92(42.6)	1.42 (0.70, 2.92)	0.330		
Mode of delivery ^b^						
Caesarean section	10(28.6)	64(29.6)	0.95 (0.43, 2.09)	0.899	0.81(0.36, 1.89)	0.639
Vaginal delivery	25(71.4)	152(70.4)	1		1	
Type of delivery ^a^						
Multiple	8(22.9)	64(29.6)	0.70 (0.30, 1.63)	0.413	0.71(0.29, 1.72)	0.450
Singleton	27(77.1)	152(70.4)	1		1	
Birth weight ^b^						
≤1500 g	21(60.0)	83(38.6)	**2.38 (1.14, 4.95)**	**0.020**	**2.36 (1.13, 4.95)**	**0.023**
>1500 g	14(40.0)	132(61.4)	1		1	
Child EBF ^b^						
Yes	11(31.4)	75(34.7)	1	1		
No	24(68.6)	141(65.3)	0.86(0.4, 1.85)	0.703	0.83(0.35, 1.95)	0.669
Duration of hospital stay ^b^						
1–7 days	12(34.3)	122(56.5)	1	1	1	
8–14 days	16(45.7)	82(38)	1.98 (0.89, 4.41)	0.093	1.84(0.81, 4.15)	0.140
15–28 days	7(20)	12(5.6)	**5.93 (1.96, 17.90)**	**0.002**	**5.77(1.88, 17.67)**	**0.002**
Duration of hospital stay ^b^						
1–7 days	12(34.3)	122(56.5)	1	1	1	
>7 days	23(65.7)	94(43.5)	**2.49 (1.18, 5.26)**	**0.017**	**2.33(1.09, 4.98)**	**0.028**
Duration in preterm clinic ^b^						
< 6 months	3(8.6)	41(19.0)	1	1	1	
6–12 months	14(40.0)	80(37.0)	2.40 (0.65, 8.80)	0.189	2.31(0.62, 8.60)	0.212
>12 months	18(51.4)	95(44.0)	2.59 (0.72, 9.28)	0.144	2.70(0.74, 9.76)	0.132
Completed one year in care ^b^						
Yes	19(54.3)	119(55.1)	1.03(0.50, 2.12)	0.929	0.95 (0.45, 0.36)	0.886
No	16(45.7)	97(44.9)	1	1		

*Reference group: Not Underweight*

*EBF: Exclusive Breastfeeding, COR: Crude Odds Ratio, CI: Confidence interval*

**Table 4:** Relationship between maternal characteristics and underweight

Variables	Nutritional status		COR 95% CI	P value
	Underweight (W/A Z-score <-2) (n = 35) (f, %)	Not Underweight W/A Z-score ≥-2) (n = 216) (f, %)		
Age				
≤ 25 years	6(17.1)	44(20.4)	0.74(0.27, 2.03)	0.56
26- 30years	16(45.7)	87(40.3)	1	1
> 30 years	13(37.1)	85(39.4)	0.83(0.38, 1.83)	0.648
Estimated Distance from home to hospital				
< 5Km	3(8.6)	25(11.6)	1	1
≥5Km	32(91.4)	191(88.4)	1.4(0.4, 4.9)	0.602
Education level				
Primary below/none	9(25.7)	42(19.4)	1.43(0.63, 3.29)	0.394
Secondary/tertiary level	26(74.3)	174(80.6)	1	1
Marital status				
Married / co-habiting	24(68.6)	175(81)	1	1
Single / divorced/ separated	11(31.4)	41(19)	1.96(0.89, 4.31)	0.096
Employment status				
Formal employment	23(65.7)	141(65.3)	1	1
Informal employment/ none	12(34.3)	75(34.7)	0.98(0.46, 2.08)	0.960
Have social support				
Yes	30(85.7)	194(90.2)	1	1
No	5(14.3)	21(9.8)	1.54(0.54, 4.39)	0.420
Number of children				
1–2 children	15(44.1)	96(45.1)	1	1
3–4 children	13(38.2)	74(34.7)	1.12(0.5, 2.51)	0.775
>5 children	6(17.7)	43(20.2)	0.89(0.32, 2.46)	0.827
Number of children				
1–2 children	15(44.1)	96(45.1)	1	
≥3 children	19(55.9)	117(54.9)	1.04(0.5, 2.15)	0.917
Participant Pregnant				
Yes	1(2.9)	18(8.3)	1.08(0.35, 3.34)	0.890
No	34(97.1)	198(91.7)	1	
On Family Planning				
Yes	19(54.3)	94(43.5)	1.54(0.75, 3.16)	0.237
No	16(45.7)	122(56.5)	1	
BMI for caretaker				
Underweight (< 18.5)	0(0)	1(0.5)		
Not underweight ≥ 18.5	34(100)	214(99.5)		
BMI for caretaker				
< 18.5	0(0.0)	1(0.5)		
18.5 to < 25	15(44.1)	91(42.3)	1	1
25 to < 30	10(29.4)	68(31.6)	0.89(0.38,2.11)	0.795
≥ 30	9(26.5)	55(25.6)	1.00(0.41,2.42)	0.987

*Reference group: Not Underweight*

**Table 5:** Relationship between infant characteristics and stunting

Variables	Nutritional status				Model 1			Model 2	
	Stunted (L/A Z-score <-2) (n = 8) (f, %)	Not Stunted L/A Z-score ≥-2) (n = 243) (f, %)	COR 95% Cl	P value	aOR 95% Cl	P-value	aOR 95% Cl		P-value
Sex of the child									
Male	64(61)	58(39.7)	**2.36(1.42,3.95)**	**0.001**	**2.39(1.42, 4.04)**	**0.001**	**2.5(1.43,4.35)**		**0.001**
Female	41(39)	88(60.3)	1		1				
Age of the child									
22–30 months	58(55.2)	83(56.8)	1		1				
31–38 months	47(44.8)	63(43.2)	1.07(0.64,1.76)	0.800	1.02(0.61,1.71)	0.944	1.03(0.6,1.78)		0.912
Mode of delivery ^b^									
Caesarean section	26(24.8)	48(32.9)	0.67(0.38,1.18)	0.165	0.70(0.38,1.23)	0.209	0.79(0.43,1.46)		0.459
Vaginal delivery	79(75.2)	98(67.1)	1		1				
Type of delivery ^a^									
Multiple	31(29.5)	41(28.1)	1.07(0.62,1.87)	0.803	1.10(0.61,2.00)	0.767	0.85(0.46,1.56)		0.6
Singleton	74(70.5)	105(71.9)	1		1				
Birth weight ^b^									
≤1500 g	47(44.8)	57(39.3)	1.25(0.75,2.08)	0.388	1.26(0.75,2.12)	0.383	1.27(0.74,2.21)		0.388
>1500 g	58(55.2)	88(60.7)	1		1				
Child EBF ^b^									
Yes	69(65.7)	96(65.8)	1		1				
No	36(34.3)	50(34.2)	1.00(0.60,1.70)	0.995	0.93(0.52,1.67)	0.817	0.92(0.52,1.63)		0.777
Duration of hospital stay ^b^									
1–7 days	54(51.4)	80(54.8)	1		1				
8–14 days	44(41.9)	54(37)	1.21	0.484	1.16(0.68,2.00)	0.587	1.28(0.72,2.28)		0.396
15–28 days	7(6.7)	12(8.2)	0.64	0.774	0.80(0.29,2.20)	0.671	1.02(0.36,2.9)		0.976
Duration of hospital stay ^b^									
1–7 days	54(51.4)	80(54.8)	1		1				
>7 days	51(48.6)	66(45.2)	1.14(0.69,1.90)	0.598	1.10(0.65,1.83)	0.729	1.24(0.71,2.15)		0.45
Duration in preterm clinic ^b^									
< 6 months	16(15.2)	28(19.2)	1		1				
6–12 months	43(41)	51(34.9)	1.48(0.71,2.04)	0.300	1.40(0.66,3.00)	0.375	1.53(0.69,3.4)		0.294
>12 months	46(43.8)	67(45.9)	1.20(0.32,2.33)	0.617	1.20(0.57,2.50)	0.641	1.24(0.56,2.72)		0.597
Completed one year in care ^b^									
Yes	54(51.4)	84(57.5)	1		1				
No	51(48.6)	62(42.5)	1.28(0.77,2.12)	0.338	1.30(0.78,2.18)	0.318	1.28(0.74,2.23)		0.379

*Reference group: Not Stunted*

*EBF: Exclusive Breastfeeding; COR: Crude Odds Ratio; CI: Confidence interval; aOR: Adjusted Odds Ratio*

**Table 6:** Relationship between maternal characteristics and Stunting

Variables	Nutritional status		COR 95% CI	Pvalue	Model 1		Model 2	
	Stunted (L/A Z-score <-2) (n = 35) (f, %)	Not Stunted L/A Z-score ≥-2) (n = 216) (f, %)	COR 95% CI	P value	aOR 95% CI	P value	aOR 95% CI	P value
Age								
≤ 25 years	30(28.6)	20(13.7)	**2.27(1.13,4.52)**	**0.020**	**2.16(1.02,4.54)**	**0.043**	1.85(0.9,3.82)	0.096
26- 30years	41(39)	62(42.5)	1		1		1	
> 30 years	34(32.4)	64(43.8)	0.80(0.45,1.43)	0.454	0.76(0.42,1.4)	0.383	0.82(0.45,1.52)	0.536
Estimated Distance from home to hospital								
< 5Km	10(9.5)	18(12.3)	1		1		1	
≥5Km	95(90.5)	128(87.7)	1.34(0.59, 3.03)	0.487	1.28(0.55,2.96)	0.570	1.31(0.54,3.16)	0.552
Education level								
Primary below/none	25(23.8)	26(17.8)	1.44(0.78, 2.67)	0.245	1.58(0.82,3.01)	0.168	1.45(0.73,2.89)	0.289
Secondary/tertiary level	80(76.2)	120(82.2)	1		1			
Marital status								
Married / co-habiting	77(73.3)	122(83.6)	1		1		1	
Single / divorced/ separated	28(26.7)	24(16.4)	**1.84(1.00,3.42)**	**0.050**	1.79(0.93,3.45)	0.079	1.58(0.81,3.08)	0.181
Employment status								
Formal employment	70(66.7)	94(64.4)	1		1		1	
Informal employment/ none	35(33.3)	52(35.6)	0.90(0.53,1.53)	0.708	0.9(0.52,1.56)	0.708	0.90(0.51,1.6)	0.718
Have social support								
Yes	93(88.6)	131(90.3)	1		1		1	
No	12(11.4)	14(9.7)	1.21(0.53, 2.73)	0.651	1.37(0.58,3.26)	0.475	1.05(0.39,2.85)	0.922
Number of children								
1–2 children	51(49)	60(42.0)	1		1		1	
3–4 children	33(31.7)	54(37.8)	0.72(0.40,1.27)	0.258	0.99(0.51,1.91)	0.973	1.02(0.51,2.05)	0.944
>5 children	20(19.2)	29(20.3)	0.81(0.41,1.60)	0.547	1.13(0.52,2.48)	0.754	1.01(0.44,2.31)	0.986
Number of children								
1–2 children	51(49.0)	60(42.0)	1		1		1	
≥3 children	53(51.0)	83(58.0)	0.75(0.45, 1.25)	0.270	1.03(0.56,1.9)	0.915	1.02(0.53,1.94)	0.954
Previous Preterm delivery								
Yes	6(6)	19(14)	**0.39(0.15,1.02)**	**0.006**	0.49(0.19,1.21)	0.122	0.42(0.16,1.13)	0.085
No	94(94)	117(86)	1		1		1	
Participant Pregnant								
Yes	8(7.6)	11(7.5)	1.10(0.42, 2.88)	0.853	0.96(0.36,2.51)	0.929	1.18(0.43,3.19)	0.750
No	97(92.4)	135(92.5)	1		1		1	
On Family Planning								
Yes	49(46.7)	64(43.8)	1		1		1	
No	56(53.3)	82(56.2)	0.95(0.56,1.60)	0.861	0.93(0.55, 1.56)	0.775	0.98(0.56,1.71)	0.935
BMI for caretaker								
Underweight (< 18.5)	0(0)	1(0.7)						
Not underweight ≥ 18.5	104(100)	144(99.3)	1		1		1	
BMI for caretaker								
< 18.5	0(0)	1(0.7)						
18.5 to < 25	50(50)	50(37)	1		1		1	
25 to < 30	30(30)	43(31.9)	0.70(0.38,1.28)	0.247	0.72(0.38,1.34)	0.300	0.75(0.4,1.39)	0.356
≥ 30	20(20)	41(30.4)	**0.49(0.25,0.94)**	**0.034**	0.64(0.32,1.29)	0.210	0.6(0.3,1.21)	0.153
Maternal Height*								
Less than 150	18(18.0)	6(4.4)	**5.00(1.79,13.99)**	**0.002**	**4.87(1.7,13.93)**	**0.003**	**4.24(1.47,12.21)**	**0.007**
150–154.9	22(22.0)	28(20.7)	1.31(0.64,2.69)	0.462	1.2(0.58,2.51)	0.622	1.27(0.61,2.65)	0.529
155–159.9	30(30.0)	50(37.0)	1		1		1	
160–164.9	23(23.0)	36(26.7)	1.06(0.53,2.13)	0.859	1.17(0.57,2.39)	0.662	1.07(0.52,2.17)	0.857
more = 165	7(7.0)	15(11.1)	0.78(0.28,2.12)	0.624	0.87(0.31,2.45)	0.787	0.69(0.25,1.93)	0.484

*Reference group: Not Stunted*

*COR: Crude Odds Ratio; CI: Confidence interval; aOR: Adjusted Odds Ratio*

## Discussion

We sought to establish the growth and nutritional status for children born with LBW at Mulago Hospital, Uganda. From our study, 101 (40.1%) of the participants had normal growth for their age while the rest of the children had undernutrition. The prevalence of stunting was 42.2% and underweight 14.4% and relatively low levels of wasting 3.2%.

Less than half of our participants had normal growth status although children born with LBW are expected to catch-up on growth as those born with appropriate birthweight at 2–3 years of life. This finding was not surprising because we studied a high-risk population. Both prematurity and low birthweight are negatively correlated to postnatal growth [23]. Secondly, undernutrition is a significant public health burden in children under-five in resource limited settings [16]. Our findings were comparable to results of the general population both globally and locally where stunting was highest among the under-fives [16, 17, 24], although these were not limited to children born with LBW. The prevalence of childhood undernutrition in the present study was higher than the country prevalence of 29% and 14.4% for stunting and underweight respectively in the recent Uganda demographic and Health Survey. Wasting was slightly lower, at 3.2% of the study participants versus 4% in the general population of under-fives [24]. We focused on early childhood because it is an important preschool period and growth impacts on learning [9, 16]. Also, the anthropometric parameters at two years can fairly predict the growth outcomes later in life [15].

In our study, 61% of the boys were stunted compared to 39% of the girls. This was documented by Zhihui et al. in several LMICs [25], although further studies are needed to evaluate the mechanism in which sex may contribute to stunting. Furthermore, there were more young mothers with stunted children compared to older mothers also shown in other studies [26, 27]. It is assumed that older mothers are knowledgeable in aspects of child care compared to young and teenage mothers [26] leading to better outcome of their children. The mothers with short stature had stunted children and this could be attributed to genetic and environmental factors [28]. Studies have demonstrated mothers with short stature or those born with low birthweight were more likely to give birth to children with the same features [15, 25]. Even though factors such as maternal education wealth quintile and maternal BMI have been described to be associated with stunting [24, 25], our study did not show the same relationship. Other factors like maternal illness e.g. diabetes, hypertension or malnutrition have been shown to affect the child’s growth. In the current study only one mother was found to have underweight while thirty-four mothers had pregnancy induced hypertension, no other chronic illness was reported.

There were more VLBW infants with underweight than those with LBW or who weighed > 1500 g. This may be explained by the difficulties encountered in feeding VLBW infants majority of whom are preterm babies, thus contributing to underweight in early childhood [29, 30]. Fortification of breastmilk and use of total parental nutrition when the LBW babies need nutritional support the most are not routinely practiced in our setting. The infants who stayed longer in hospital were likely to have underweight later in life. This could be an indicator of difficult in feeding or generally ill-health which may hinder adequate feeding and growth. Only 3.2% of our study participants were wasted as compared to the 4% in the general population [24]. Wasting is an indicator of acute illness and we did not identify children in whom recent acute illness was reported and this probably would explain the low prevalence.

Our study findings show that undernutrition is higher in this at-risk population and growth monitoring should extend to childhood and beyond. Child growth and nutritional status may be strongly linked to fetal life suggesting a need for interventional focus on nutrition during pregnancy and early childhood [15]. To end all forms of malnutrition by 2030: Sustainable Development Goal 2 [31], a life-course approach of nutritional interventions are needed to break the vicious cycle of health problems related to undernutrition such as LBW. These will in turn lead to child survival, educational achievements and overall well- being later in life. Secondly, collection and analysis of long-term data in former LBW children linked to nutritional strategies and growth parameters are strongly recommended in our setting. Although pre-pregnancy and natal nutritional status was not assessed in our study, they have been linked to growth failures in early childhood in other studies [23, 28]. It is therefore important to mind the mother’s nutritional status from pre-conception throughout pregnancy [15, 16].

The strength of this study was the predominant mode of feeding was exclusive breastfeeding with no modification for all the participants. The results are therefore generalizable to settings where fortification of preterm feeds is not readily available. There is limited literature on the topic in our setting, our study provides additional evidence to guide interventions aimed at improving outcome children born with LBW. The limitations of a cross-sectional design were data not reflecting changes in growth of individual children overtime and inferring cause of LBW and undernutrition among the participants. This was a single center study, a national referral hospital with variations in the clients served. The fairly small sample size affected the power to analyze for association of LBW and undernutrition. Nevertheless, the results clearly indicate a need for rigorous growth monitoring for children born LBW beyond the neonatal period.

## Conclusion

Six of every ten children born with LBW are at risk of undernutrition in early childhood: underweight and stunting were highly prevalent compared to the national prevalence. Targeted interventions are specifically needed for children born with very low birthweight, males and those requiring long postnatal hospitalization.

## Data Availability

All data and materials supporting the conclusions of this article are included within the manuscript. The datasets used and/or analyzed during the current study are available from the corresponding author on reasonable request.

## Abbreviations

HAZ: Height for-age z-scores
LBW: Low birth weight
LMICs: Low- and Middle-income Countries
WAZ: Weight-for-age z-scores
WHZ: Weight-for-height z-scores
WHO: World Health Organization
